# Biodegradation of Pine Processionary Caterpillar Silk Is Mediated by Elastase- and Subtilisin-like Proteases

**DOI:** 10.3390/ijms232315253

**Published:** 2022-12-03

**Authors:** Alba Diez-Galán, Rebeca Cobos, Ana Ibañez, Carla Calvo-Peña, Juan José R. Coque

**Affiliations:** Instituto de Investigación de la Viña y el Vino, Escuela de Ingeniería Agraria, Universidad de León, 24009 León, Spain

**Keywords:** silk, pine processionary, *Bacillus*, *Pseudomonas*, biodegradation, proteases, elastase, subtilisin

## Abstract

Pine processionary caterpillar nests are made from raw silk. Fibroin protein is the main component of silk which, in the case of pine processionary caterpillar, has some unusual properties such as a higher resistance to chemical hydrolysis. Isolation of microorganisms naturally present in silk nests led to identification of *Bacillus licheniformis* and *Pseudomonas aeruginosa* strains that in a defined minimal medium were able to carry out extensive silk biodegradation. A LasB elastase-like protein from *P. aeruginosa* was shown to be involved in silk biodegradation. A recombinant form of this protein expressed in *Escherichia coli* and purified by affinity chromatography was able to efficiently degrade silk in an in vitro assay. However, silk biodegradation by *B. licheniformis* strain was mediated by a SubC subtilisin-like protease. Homologous expression of a subtilisin Carlsberg encoding gene (*sub*C) allowed faster degradation compared to the biodegradation kinetics of a wildtype *B. licheniformis* strain. This work led to the identification of new enzymes involved in biodegradation of silk materials, a finding which could lead to possible applications for controlling this pest and perhaps have importance from sanitary and biotechnological points of view.

## 1. Introduction

Silk is one of the most abundant naturally derived polymers. In fact, silk fibers are a common material naturally produced by a wide variety of arthropods that use them to build their cocoons (silkworms), webs (spiders) and nests (honeybees, wasps, and butterflies, among others) [1]. Silk production by the *Bombyx mori* silkworm has developed into a major textile industry producing around 120,000 metric tons of silk per year, with the primary manufacturers located in China, India and Japan [1]. We know that most of the 113,000 known species of *Lepidoptera* can produce silk [2]. One species of silk-producing lepidopteran is the pine processionary moth (*Thaumetopoea pityocampa*). This nocturnal lepidopteran is of particular interest because it has become one of the most harmful pests affecting forests of several *Pinus*, *Cedrus* and *Pseudotsuga* species in many countries around the Mediterranean basin. Pine processionary threatens the ecology and sustainability of pine and cedar forests in many countries in central-southern Europe and North Africa [3,4]. The problem is worsening because in recent years, and as a putative consequence of global warming, a latitudinal and altitudinal expansion of the pine processionary has been reported in Europe. As a consequence, this pest is moving to the north of Europe and it has already been detected in Switzerland and Germany [5], threatening their forests in geographical areas hitherto free of this pest. Furthermore, this species is of interest because of the severe allergic reactions it can cause in humans, domestic pets and other animals, even resulting in death, which increases the interest in its control [6].

In winter the pine processionary larvae feed voraciously on coniferous needles, causing severe defoliation which results in both a decrease of the tree’s growth rate and a decrease in its annual diameter increment.

Furthermore, defoliated trees can become highly prone to attack by other insects and pests, resulting in a higher mortality rate in the affected forests [7]. Typically, in winter the larvae build and live inside silk nests which they leave during the night for feeding on pine foliage. During the day the larvae rest within these large communal “nests” constructed of silken filaments. These nests are firmly attached to the branches of the host tree and accommodate 50 or more caterpillars and contain a lot of debris in the form of broken pine needles, wood fragments, fecal matter, and body hairs shed by the caterpillars. The nests usually have thick, resistant silk walls, providing an additional protection to the larvae against weather conditions, potential predators and chemical treatments. 

Natural silk is characterized by high strength, elasticity, thermal insulation and hygroscopicity (in natural conditions silk contains approximately 11% moisture, and it can have a moisture content of 30% without feeling wet). Silkworm raw silk consists of protein nanofibers–fibroins–stuck together in groups of 2. Sericin protein envelops the nanofibers and acts as a glue that holds them together [8,9]. Fibroin makes up 70–75% of raw silk whereas sericin represents around 25–30%. Sericin is a protein composed of 18 different amino acids of which serine is the most abundant, representing around 32% of the total amino acids. Silkworm fibroin is composed of two protein chains, heavy-chain (H-fibroin) with a molecular weight of approximately 350 kDa and light chain (L-fibroin, Mw~26 kDa) covalently linked by a disulfide bond at the carboxy-terminus of the two subunits. Disulphide bridges are responsible for fiber strength. There are also hydrogen bonds within and between the molecules. The complete amino acid sequence of the *B. mori* fibroin heavy chain is composed of a highly repetitive (Gly-Ala)n sequence motif and tyrosine-rich domains [8,9]. In fact, around 90% of it consists of four amino acids: alanine, glycine, serine, and tyrosine [10]. 

Basic characterization of silk fibroin from pine processionary was carried out a long time ago by Shaw and Smith [11], indicating some typical traits as compared to other silk fibroins, such as a higher content of amino acids with hydrophilic side-chains, or a higher resistance to acid hydrolysis. Unfortunately, no further studies on this type of silk have been carried out to date. 

Microbial degradation of other types of silk, particularly silkworm silk (*B. mori*), is well documented. Strains of *Burkholderia cepacia* [12] and *Variovorax paradoxus* [13] are able to biodegrade silkworm silk. Microorganisms probably assimilate sericin more easily than fibroin and it is known that decomposition of sericin primarily involves proteolytic enzymes [13]. Two unidentified species of bacteria (belonging to the *Streptomyces* genus) have also been reported to produce proteases hydrolyzing a variety of insoluble proteins, including silk [14,15]. In vitro tests have also confirmed the degradation of fibroin by protease XIV from Streptomyces griseus [16] and protease XXI from *Streptomyces* sp. [17]. Both proteases are actually a mix of proteolytic enzymes including at least three different proteases, of which at least one is an extracellular serine protease. Unfortunately, no information is available in the literature indicating the microbial degradation of pine processionary silk.

Current control strategies employed to suppress the populations of this detrimental pine tree pest are primarily based on treatments with chemical pesticides and biopesticides, natural compounds and predators, or biocontrol agents, and also the removal of egg packages, enhancement of natural enemies’ activities, and destruction of winter nests made of silk [4,18]. In this context, any putative technology that contributes to the degradation and/or disorganization of the nests could be an effective aid in the control of this plague. This work addresses the isolation and characterization of bacteria with the ability to degrade raw pine processionary silk and identification of some proteases involved in the biodegradation process.

## 2. Results

### 2.1. Structure of Pine Processionary Raw Silk

**Figure 1:**
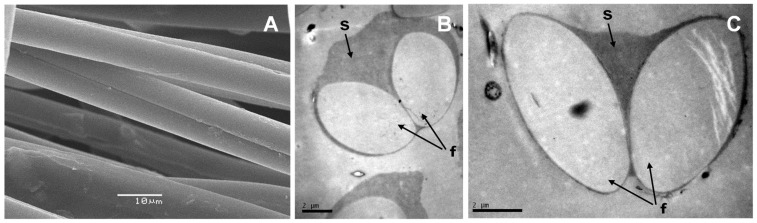
Scanning electron microscopy (SEM) visualization of fibers of raw pine processionary silk (×2000) (**A**). The basic unit is formed by 2 fibroin nanofibers arranged in parallel and laterally associated with other fibers of the same nature (**A**); cross sections of silk basic units observed by transmission electron microscopy (TEM) at 6000 magnification (**B**) and 10,000 magnification (**C**). Note the sericin layer (s) acting as a glue that holds the fibroin nanofibers (f) together.

Electron microscopy analysis confirmed that the basic structure of raw pine processionary silk consists of 2 long fibroin nanofibers running in parallel (Figure 1A) which are associated to form a more complex network of parallel fibers. Both fibroin fibers are held together by a sericin layer that acts as a glue (Figure 1B,C), with a structure very similar to that described for silkworm silk. The cross-section of the nanofibers is ovoid and their average dimensions are 8–10 μm × 4–5 μm (Figure 1B,C).

### 2.2. Amino Acid Analysis of Silk

Acid hydrolysis of raw silk allowed the detection of 9 amino acids, listed in decreasing concentration: glycine (14.85 ± 0.42 mM), serine (9.19 ± 0.10 mM), alanine (5.72 ± 0.33 mM), aspartic acid (5.68 mM ± 0.24 mM), glutamic acid (2.14 ± 0.14 mM), tyrosine (1.64 ± 0.08 mM), arginine (1.37 ± 0.03 mM), leucine (1.32 ± 0.11 mM) and valine (0.87 ± 0.05 mM). Some traces of cysteine and phenylalanine/isoleucine mixture (both amino acids coeluted with the chromatographic conditions used) were also detected although they could not be quantified. 

### 2.3. Isolation of Silk-Degrading Bacterial Strains

In the initial experiment carried out in a poorly defined minimal medium to force silk biodegradation, no obvious signs of degradation were visually observed during the first 60 days of incubation. Around day 75 some evident degradation and disorganization of the silk ball began to be observed, which became more evident around day 90 of incubation, when only a few loose silk threads remained in suspension. In order to enrich the sample in putative silk degrading microorganisms, a new batch of silk was reinoculated with 0.5 mL of the initial culture under the same conditions as the initial isolation but including glucose at a final concentration of 0.1%. This enrichment step was repeated twice. In this case we observed a clear acceleration of the degradation process which was completed on average 10–12 days after inoculation of the sample. Plating serial dilutions of the enrichment cultures on PCA allowed us to isolate a total of 5 different bacterial strains based on easily observable microscopic and macroscopic traits of the colonies. 

A new sterile silk degradation experiment was performed for each of the isolated bacteria in pure culture, and we observed that only 2 out of the 5 isolated bacteria showed clear silk degradation capability. Partial sequencing of their 16S rDNA allowed us to identify these isolates as *Pseudomonas aeruginos*a IIVV-SD1 and *Bacillus licheniformis* IIVV-SD3 (Supplementary material Figure S1). Strains were named as IIVV by “Instituto de Investigación de la Viña y el Vino”, the research center in which they were isolated and named as SD referring to their “Silk Degrading” capability.

**Figure 2:**
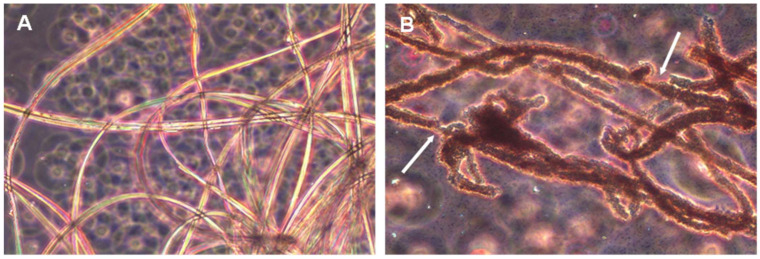
Observation of a *P. aeruginosa* pure culture under optical microscope (40×) in the presence of raw silk. (**A**) 12 h of incubation; (**B**) 48 h of incubation. Note the intense bacterial colonization observed on the surface of silk fibers in the 48-hour culture, where silk biodegradation was already apparent. At some points fibers experienced a considerable decrease in thickness (points marked with arrows), which could cause the fibers to break into shorter fragments.

Both strains were able to achieve extensive biodegradation of silk in minimal medium, which indicated that silk could be used as both a carbon and nitrogen source. The observation of liquid cultures under optical microscopy revealed that both isolates with degradation capability were able to intensively colonize and attach to the surface of the silk fibers (Figure 2A), producing an evident disorganization and degradation of the fibers. At some points fibers experienced a considerable decrease in thickness, which could even cause them to break into shorter fragments.

**Figure 3:**
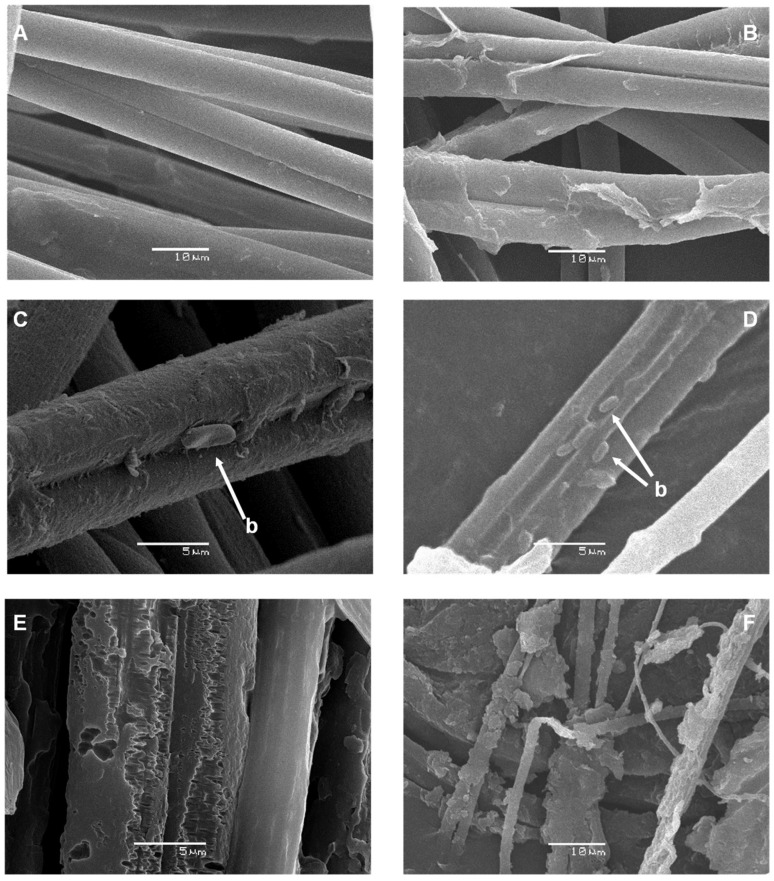
Scanning electron microscopy (SEM) visualization of raw pine processionary silk filaments and their successive degradation over time mediated by a liquid culture of *B. licheniformis*. Silk native structure at 0 h of incubation (×2000) (**A**); partial removal of the sericin surface layer observed at 24 h of incubation (×2000) (**B**); almost complete elimination of the sericin layer detected at 36 h of incubation and presence of some bacterial cells (b, indicated by arrows) attached to the underlying fibroin fibers (×5000) (**C**) (×3000) (**D**); extensive degradation with loss of fibroin fiber structure observed after 48 h of incubation (×5000, (**E**)) (×2000, (**F**)).

Silk biodegradation was confirmed by SEM visualization (Figure 3). Silk biodegradation seemed to start with removal of the outer sericin layer (Figure 3C,D). After that, bacterial cells could gain access to the inner fibroin structure to achieve extensive biodegradation with concomitant loss of fiber structure (Figure 3E,F).

### 2.4. Analysis of Silk Biodegradaton by B. licheniformis and P. aeruginosa in Liquid Media

**Figure 4:**
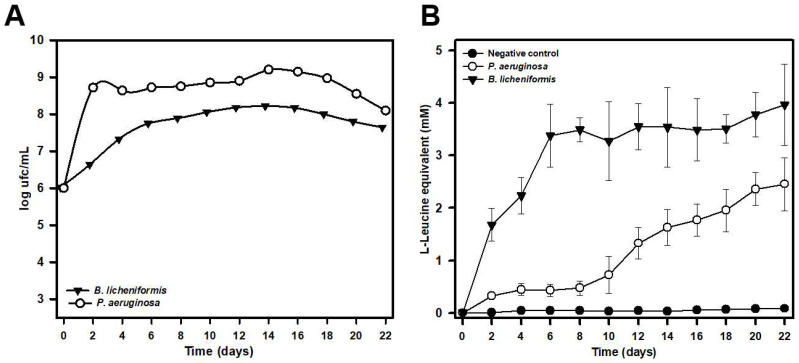
Growth rate of *P. aeruginosa* IIVV-SD1 and *B. licheniformis* IIVV-SD3 strains in a minimal medium supplemented with raw silk (**A**), and biodegradation of pine processionary raw silk estimated by release of L-Leucine equivalent (mM) into the culture medium (**B**). Bacterial cultures were run in parallel to a negative control of raw silk incubated in a non-inoculated minimal medium. The results shown are the average of two different experiments performed in triplicate.

The ability of both *B. licheniformis* IIVV-SD3 and *P. aeruginosa* IIVV-SD1 strains to biodegrade silk in liquid media was tested in in vitro experiments run for 30 days (Figure 4) in a minimal medium containing sterile silk. Under these experimental conditions we observed the release of amino acids into the culture supernatant, indicating that both bacterial strains were able to biodegrade silk. *B. licheniformis* growth proceeded quickly over the first 2 days of the experiment reaching a maximum growth around day 14 with an average of 1.13 × 10^8^ cfu/mL (Figure 4A). The amino acid release by *B. licheniformis* increased rapidly during the first 6 days of the experiment, later increasing more slowly until around day 22. From that day on, the concentration of amino acids in the culture supernatant tended to remain constant (Figure 4B).

The behavior of *P. aeruginosa* showed some remarkable differences. Growth under the experimental conditions used was slower and more sustained during the first 12 days of the experiment, reaching maximum growth around day 16, with a mean value of 1.58 × 10^9^ cfu/mL, an order of magnitude higher than that detected for *B. licheniformis* (Figure 4A). However, despite faster *P. aeruginosa* growth, silk degradation, and therefore the release of amino acids into the culture supernatant, was slower. It increased slowly and steadily during the first 10 days of incubation, increasing more rapidly between days 10 and 20. Beyond day 20, the concentration of amino acids in the supernatant tended to stabilize (Figure 4B).

### 2.5. Genome Analysis of B. licheniformis IIVV-SD3: In Silico Analysis to Identify Putative Proteases Involved in Silk Biodegradation

The draft genome sequence of *B. licheniformis* IIVV-SD3 presented an estimated genome size of 4,391,155 bp representing a coverage of 92.65% of the reference genome. It contained 5440 protein-coding genes, of which 4222 were annotated (77.61%). Of the annotated genes, functional annotation of the GO terms was found for 3647 (86.38%) and of InterPro terms for 3973 (94.1%) genes. 

In an attempt to identify putative enzymes involved in silk degradation we focused our attention on the detection of genes encoding proteases/peptidases with an extracellular or cell wall location. Prediction of their putative cellular localization was very interesting since we could assume that silk-degrading proteases would be mostly extracellular or associated to cell wall.

Thus, a total of 6 peptidases had a possible cell wall localization whereas 20 peptidases were classified as extracellular (Supplementary material Table S1). Of these, a total of 6 appear to be peptidases associated with wall peptidoglycan processing; most were Ser-type D-Ala-D-Ala-carboxypeptidases (proteins number 5, 6 and 20 in Table S1), and Cys peptidoglycan endopeptidases (proteins 11, 12 and 15. Table S1) and therefore they were initially discarded as possible candidates involved in silk degradation. Other candidates were discarded according to their biological activity (Glutathione hydrolase proenzyme; protein number 1), or their minor character (Proteins 4 and 14—Minor extracellular proteases Epr; protein 9—minor extracellular protease vpr). Among the remaining extracellular proteins, protein 13 (subtilisin Carlsberg Ser-endopeptidase) particularly attracted our attention because it is an extracellular alkaline serine protease, catalyzing the hydrolysis of proteins and peptide amides that shows high specificity for aromatic and hydrophobic amino acids at the P1 position of the substrate [19,20]. According to Shaw and Smith [11], and our own results, aromatic and hydrophobic amino acids are abundant in pine processionary fibroin and therefore this protein may contain a high number of potential cleavage sites that would allow for its efficient degradation. Subtilisin Carlsberg also exhibits a number of other attractive properties, such as high thermostability, wide range of pH compatibility, and broad specificity [20], that make it an ideal candidate for its putative involvement in silk degradation, so we decided to focus on it for further studies. 

A single copy of a gene encoding a subtilisin Carlsberg was detected in the genome of *B. licheniformis* IIVV-SD3 strain (see Supplementary material Table S2 for the nucleotide sequence of the *sub*C gene and the corresponding amino acid sequence of SubC protein). Homologous genes are called *sub*C (from subtilisin Carlsberg), *apr_2* or *apr* (from alkaline protease), or *apr*E (from alkaline protease E or subtilisin E) by different authors. We decided to adopt the terminology suggested by the Uniprot database (https://www.uniprot.org/uniprotkb/P00780/entry accessed on 24 October 2022), accepting the designation *sub*C (and using *apr* as its synonym). Thus the *B. licheniformis* IIVV-SD3 strain *sub*C gene encoded a protein of 379 amino acids with a molecular weight of 38,860 Da and an 8.73 isoelectric point. The corresponding SubC protein exhibits a 100% amino acid identity with other *B. licheniformis* proteins annotated as keratinase (AFT92040.1) and S8 family peptidase (WP_025808265.1), and slightly lesser identities (99.47% to 99.74%) with several other proteins annotated as subtilisin Carlsberg (P00780.2, QNT35445.1), protease (ABU68339.1), keratinase (AAS86761.1), or S8 family peptidase (WP_186442051.1).

### 2.6. Genome Analysis of P. aeruginosa IIVV-SD1: In Silico Analysis to Identify Putative Proteases Involved in Silk Biodegradation

The draft genome sequence of *P. aeruginosa* IIVV-SD1 presented an estimated genome size of 6,378,951 bp representing a coverage of 92.27% of the reference genome. It contained 5988 protein-coding genes, of which 4463 were annotated (74.53). Of the annotated genes, functional annotation of the GO terms was found for 4192 (93.93%) and of InterPro for 4364 (97.78%) genes. 

To identify putative enzymes involved in silk degradation, again we were interested in predicting their putative cellular localization since we assumed that the proteases responsible for silk degradation should be mostly extracellular or cell wall-associated. A total of 25 peptidases had a possible cell wall localization, perhaps located at the level of the outer cell membrane and/or in the periplasmic space. A total of 9 peptidases were classified as extracellular (Supplementary material Table S3). Among them two proteins caught our attention, namely the metalloendopeptidases LasA (protein 6 in Table S3) and LasB (protein 2 in Table S3). The latter, also known as elastase or pseudolysin, was particularly interesting since it is a widely studied protease with broad specificity although it favors hydrophobic or aromatic amino acid residues, with preference to Phe and Leu at the P1′ position. LasB also cleaves proteins with Gly at the P1 position, such that the preferred cleavage sequence is P1Gly-P1′(Leu/Phe)-P2′Ala [21]. Interestingly, porcine elastase exhibits a different cleavage pattern, mainly cutting after Ala, Gly, Leu, Ile, Ser and Val residues [22]. As we have previously indicated, according to Shaw and Smith [11] and our own results, Gly, Ser and Ala are highly abundant, with Leu and Phe present in a lower amount, in raw pine processionary silk and therefore silk proteins may contain a high number of potential cleavage sites that allow for efficient degradation by both *P. aeruginosa* and porcine elastases. *P. aeruginosa* LasB elastase is the predominant protease in the *P. aeruginosa* secretome and is an important virulent factor [23]. Moreover, porcine elastase was commercially available to perform some preliminary degradation tests, so we decided to focus on this protease for further studies. 

A single copy of a *las*B gene encoding elastase (LasB protein) was detected in the genome of *P. aeruginosa* IIVV-SD1 strain (Supplementary material Table S2 for the nucleotide sequence of the *las*B gene and the corresponding amino acid sequence of LasB protein). It encodes a protein of 498 amino acids with a molecular weight of 53,687 Da and a 5.99 isoelectric point. The corresponding LasB protein exhibits an amino acid identity ranging between 98.59% and 100% (WP_058140091.1) with many different proteins annotated as M4 family elastase LasB proteins from other *P. aeruginosa* strains. 

### 2.7. In Vitro Silk Biodegradation by Commercial Enzymes

**Figure 5:**
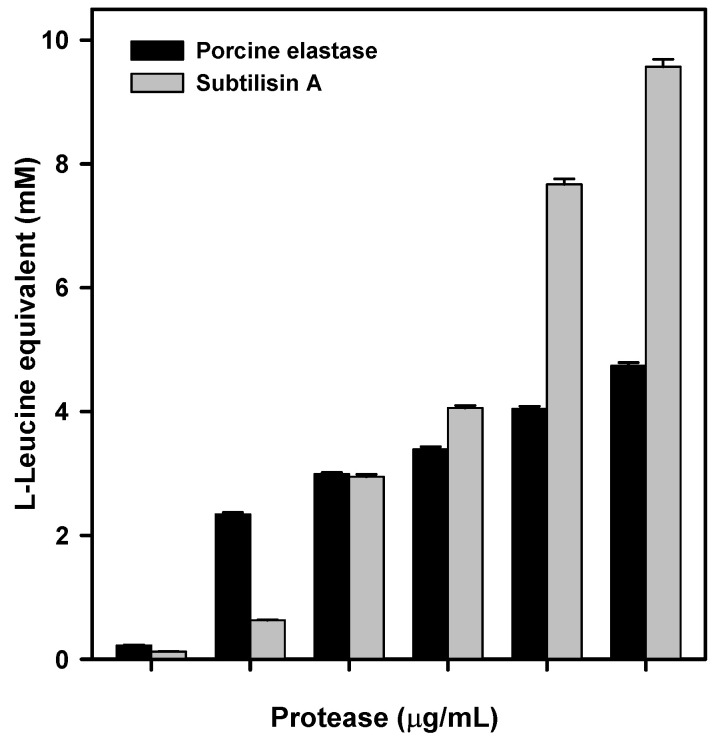
L-Leucine equivalent (mM) release after incubation (37 °C/24 h) of raw silk with different amounts of the commercial protease porcine elastase and *B. licheniformis* subtilisin A. The results shown are the average of two different experiments performed in triplicate.

Both commercial enzymes, porcine elastase and subtilisin A from *B. licheniformis*, were able to digest raw silk when incubated at 37 °C for 24 h, producing a significant release of amino acids in the reaction medium (Figure 5). Clear and visually evident degradation of the silk ball was also detected in the reaction tubes at the end of the incubation. In fact, the silk ball disappeared almost completely and only a few small, isolated filaments could be observed as a result of the degradative process. Degradation rate was dependent on enzyme concentration and for the highest concentrations (50 and 100 μg/mL) we observed that subtilisin produced a greater silk degradation than elastase, as evident from the higher amount of L-Leucine equivalents released into the reaction solution. 

### 2.8. Heterologous Expression of a P. aeruginosa LasB Elastase-Encoding Gene in E. coli and Its Role in Silk Biodegradation

**Figure 6:**
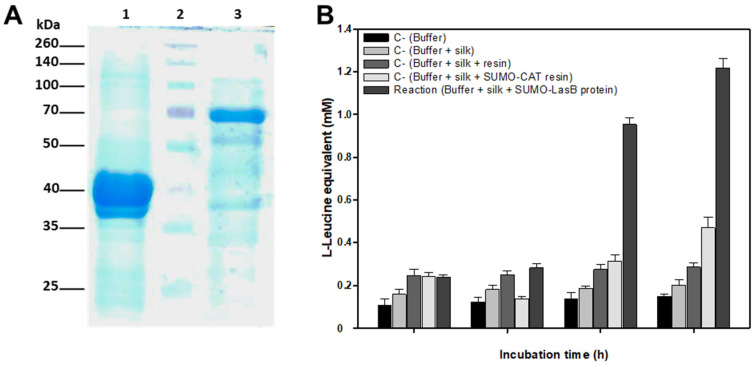
(**A**) Expression in *E. coli* and purification of SUMO-LasB recombinant protein by Ni-NTA affinity chromatography. Lanes: recombinant SUMO-CAT protein bound to Ni-NTA resin (1); Spectra multicolor Broad Range Protein Ladder (Thermo Fisher Scientific, Waltham, MA, USA) (2); recombinant SUMO-LasB protein bound to Ni-NTA after refolding (3). (**B**) Raw silk enzymatic degradation by SUMO-LasB recombinant protein as compared with different negative controls (C-) including incubation of silk in the presence of a recombinant SUMO-CAT protein attached to a Ni-NTA resin. The results shown are the average of two different experiments performed in triplicate.

A recombinant LasB-SUMO protein was efficiently expressed in *E. coli* using pET SUMO protein expression system (Figure 6A). Despite the fact that this system was designed to produce soluble fusion proteins with a small ubiquitin-like modifier (SUMO) to allow expression, purification, and generation of soluble recombinant proteins in *E. coli*, in our case the recombinant protein was insoluble and was located in inclusion bodies. In order to try to increase the production of soluble recombinant proteins, different modifications were attempted, such as induction using lower concentrations of IPTG, growth at 30 °C, or growth in a poorer culture medium or without agitation. However, all tests attempting to increase the solubility of the recombinant protein were unsuccessful. For that reason, we had to solubilize the inclusion bodies using high concentrations of guanidine hydrochloride. Once solubilized, the recombinant protein was purified on a Ni-NTA resin and directly refolded, attached to the resin using successive washing steps with buffers containing decreasing amounts of guanidine hydrochloride until a final wash with a guanidine hydrochloride-free refolding buffer (Figure 6A).

The refolded recombinant protein had an approximate molecular weight of 67 kDa as estimated by SDS-PAGE gel which corresponds fairly closely to the sum of the molecular weights of SUMO (13 kDa) and LasB (53.687 kDa) (Figure 6A; lane 3). The incubation of raw silk with the refolded recombinant SUMO-LasB protein produced the release of L-Leucine equivalent into the reaction medium, with the release being most evident at 6 and 12 h of incubation (Figure 6B). Different negative controls were performed in parallel to ensure that the release of amino acids was due to enzymatic action of the recombinant protein on the silk (Figure 6B). As it can be seen in Figure 6A, lane 3, after the refolding of the recombinant protein some bands of smaller molecular size were detected in the gel. These bands could correspond to fragments obtained by the partial degradation of the recombinant protein. In fact, we cannot rule out that in the samples analyzed there may be traces of proteases responsible for this partial degradation. The release of a small amount of L-Leucine equivalent observed in the negative control SUMO-LasB protein (Figure 6B), which tends to increase with incubation time, could be due to the existence of the previously mentioned proteases.

### 2.9. Overexpression of a B. licheniformis Subtilisin Carlsberg-Encoding Gene Enhances Silk Biodegradation

Although a similar strategy to that employed for the *P. aeruginosa* LasB protein was tried for expression of a recombinant SUMO-subtilisin protein, all attempts were unsuccessful. Therefore, a different strategy was chosen which consisted in the homologous expression in *B. licheniformis* IIVV-SD3 strain of a *sub*C gene, encoding a subtilisin Carlsberg isolated from that same strain. 

Two different transformants (B1 and B2) carrying the recombinant plasmid pHY300PLK-SubC, which includes a copy of the *sub*C gene under the control of its own promoter region, were analyzed to determine its number of copies. Thus, the amplification specificity of *sub*C and *rpo*B genes was checked by melting curve analysis (Supplementary material Figure S2A). The *sub*C primer set showed a sharp peak for the PCR product of the quantitative standard sample at 84.38 ± 0.02 °C. A single melting peak at the same melting temperature was produced for the PCR product of the total DNA sample. The *rpo*B primer set showed a single melting peak for the quantitative standard as well as for the total DNA sample at the same temperature of 77.58 ± 0.02 °C. Every PCR product also yielded prominent bands with expected sizes of 100 and 70 bp, respectively, after gel electrophoresis analysis. The identity of amplified products was additionally confirmed by DNA sequencing. These results indicated that non-specific PCR products were not detected in the analyzed temperature range with the primer sets used (Supplementary material Figure S2A). The ratio between mass and copy number was calculated for each PCR product resulting in 1 ng corresponding to 9.26 × 10^9^ copies for *sub*C PCR product and 1.32 × 10^10^ copies for *rpo*B PCR product. The standard curves for *sub*C and *rpo*B each ranged from 1 × 10^6^ to 1×10^10^ copies (Supplementary material Figure S2B) obtained. Both curves were linear in the range tested (*R*2 > 0.998) in the triplicate reactions. The slopes of the standard curves for *sub*C and *rpo*B were −3.755 and−3.061, respectively, with an amplification efficiency of 84.1% for *sub*C and 107.6% *rpo*B. Standard curves were used for relative quantification. For relative quantification, *sub*C and *rpo*B were used as the target and reference genes, respectively, and the plasmid copy number was determined by the 2^−ΔΔ*C*T^ calculation (Supplementary material Figure S2C). Since *B. licheniformis* only has one copy of the *rpo*B gene in its genome, the relative quantification showed that both *B. licheniformis* IIVV-SD3 transformants analyzed harboring the pHY300PLK-*sub*C plasmid contained, in the middle of the exponential phase, an average of 7 more copies of the *sub*C gene than the *B. licheniformis* IIVV-SD3 wild type and accordingly, the average plasmid copy number in each transformant was 7.

**Figure 7:**
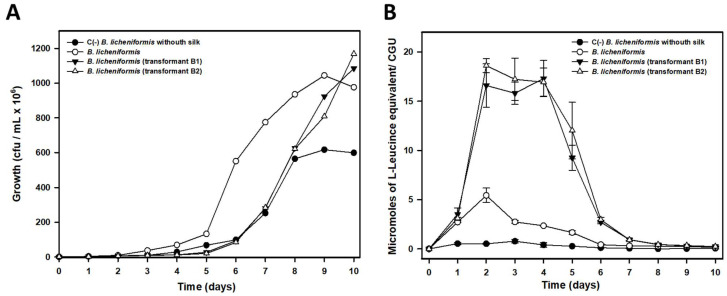
Stimulation of raw silk biodegradation in *B. licheniformis* B1 and B2 transformants carrying 8 copies (1 chromosomal copy and 7 plasmid copies) of the *sub*C gene compared to the biodegradation rate observed in the wild-type strain IIVV-SD3 carrying a single chromosomal copy of the *sub*C gene. (**A**) Growth rate estimated as viable cells or cfu/mL. (**B**) Release of L-Leucine equivalent (µmol) into the culture media from silk biodegradation. Given the different growth rates observed in the transformants compared to the wild-type IIVV-SD3 strain, the results were normalized to the number of viable cells (cfu) detected in the cultures and expressed as CGUs (Cellular Growth Units). One CGU equals 1 × 10^7^ viable cells. The results shown are the average of two different experiments performed in triplicate.

As seen in Figure 7A both transformants exhibited a delayed growth rate as compared to that of *B. licheniformis* growth in the presence of raw silk. This delay in growth could be due, among other factors, to the presence of the antibiotic tetracycline added to the culture medium as a selective pressure to favor the maintenance of the plasmid carrying the additional copies of the *sub*C gene, or some growth interference due to the plasmid.

Therefore, in order to be able to compare the results of amino acid release into the culture medium from silk biodegradation, the normalized results are shown in Figure 7B, as a function of the number of viable cells present at each moment in the culture. Thus we can see how, with equal numbers of cells, both B1 and B2 transformants, carrying a total of 8 copies of the *sub*C gene (1 chromosomal copy and 7 plasmid copies), showed an enhanced silk biodegradation capacity between days 2 and 6 of the experiment. More precisely, by day 4 of culture, transformant B1 exhibited a biodegradative capacity (estimated as a function of the levels of amino acids present in the culture supernatant) that was 7.36 times higher than that observed in the wild-type strain. In the case of transformant B2, the biodegradative capacity was 7.20 times higher. This result confirmed that the transformants carrying additional copies of the *su*bC gene were able to perform more efficient silk biodegradation compared to the wild-type strain. At the end of the experiment, growth of both transformants in the presence of silk was 1.63–1.95 times greater than that observed in the wild type strain, most likely due to utilization of the released amino acids as a source of carbon, nitrogen and energy.

## 3. Discussion

Despite the importance of the pine processionary as a forest pest and the significant allergic reactions it causes, there are few studies on its raw silk. To our knowledge only Shaw and Smith [11] analyzed pine processionary silk in an old work to conclude that its fibroin had some unusual properties including a higher content of amino acids with hydrophilic side-chains, and also an increased resistance to acid hydrolysis as compared to silk from Tiger moth (*Arctia caja*) and Oak Eggar (*Lasiocampa quercus*). Unfortunately, these authors did not use silkworm silk, which is certainly the most studied, in their study.

According to our work, there are many similarities between pine processionary and silkworm silk. Electron microscopy analysis shows that pine processionary silk has a very similar macrostructure compared to that of silkworm silk. Thus, as in the case of silkworm silk, each fiber contains 2 fibroin filaments coated with an outer glue-like coating, probably made of sericin [1]. Amino acid analysis of raw pine processionary silk indicated a predominance of non-polar hydrophobic amino acids such as glycine, alanine, valine, leucine, and tyrosine, and polar hydrophilic amino acids including neutral (serine) and acidic (aspartic and glutamic acid) amino acids. This amino acid composition was very similar to that observed by Shaw and Smith [11] who reported that the 3 most abundant amino acids after hydrolysis of pure fibroin were glycine, serine, and alanine, exactly the same ones that appear in our analysis. Small differences in amino acid composition could be due to the different analytical methods used and also to the fact that whereas Shaw and Smith analyzed pure fibroin, we analyzed raw silk (sericin plus fibroin). In a different study, Do and colleagues [24] reported that the most abundant amino acids in silkworm fibroin pretreated to remove sericin and other impurities were glycine (40.4%), alanine (30.1%) and serine (10.2%). More recently Bungthong and colleagues [25] analyzed the amino acid profile of silkworm silk after water and enzyme extraction, and although the methodology is totally different and therefore the results cannot be easily compared, again glycine was the most abundant amino acid with both serine and alanine also particularly abundant. Therefore, we can conclude that pine processionary silk and silkworm silk have a very similar amino acid composition, with a predominance of non-polar hydrophobic amino acids (glycine, alanine) and a neutral hydrophilic amino acid like serine.

Since silk is one of the most abundant naturally derived protein polymers it should come as no surprise that there are numerous microorganisms and mechanisms involved in its biodegradation. The isolation from silk nests in an advanced state of degradation of *B. licheniformis* and *P. aeruginosa* strains with the ability to biodegrade silk confirmed this fact. The biodegradative capacity was confirmed by studies with pure cultures in minimal liquid medium indicating that these microorganisms can use the peptides and amino acids released in the degradation process as a source of carbon, nitrogen, or energy. Analysis by both optic and electron microscopy also confirmed that these microorganisms are able to degrade silk to the point of completely destroying the native structure of the filaments. The biodegradative capability of *B. licheniformis*, in the experimental conditions tested, was higher than that observed for *P. aeruginosa*, as deduced from the higher concentration of L-Leucine equivalent released into the culture media, even at the same or a lower number of cells. This higher biodegradative capacity could also be visually verified since *B. licheniformis* cultures did not leave any silk filament remnants in liquid cultures, while small filaments could be observed in the case of *P. aeruginosa*, which also suggested a lower biodegradative capacity of this species. The higher biodegradation capability of *B. licheniformis* compared to that observed by *P. aeruginosa* could be due to the different cleavage specificity of subtilisin and elastase proteins. In fact, and according this different cleavage specificity, a much larger number of potential cleavage sites for subtilisin should exist in pine processionary fibroin since this protease catalyzing the hydrolysis of proteins and peptide amides shows high specificity for aromatic and hydrophobic amino acids at the P1 position of the substrate [19,20]. These amino acids are particularly abundant in pine processionary fibroin according to Shaw and Smith [11], and our own results.

Microbial degradation of silkworm silk by both bacterial and fungal strains had been previously reported [10]. Regarding bacterial degradation, Seves and colleagues [12] isolated a *Burkholderia cepacia* strain able to use fibroin as a sole source of carbon and nitrogen for growth. A *Variovorax paradoxus* strain was also able to grow in a minimal medium containing silk fibroin as the sole source of carbon and nitrogen [13]. A silk-degrading enzyme, called fibroinase, was partially purified. The enzyme had a 21 kDA molecular weight and was also able to degrade casein and, to a smaller extent, collagen and albumin, although more accurate identification of this enzyme was not achieved. More recently *B. subtilis* and *P. fluorescens* were isolated by their ability to degrade sericin [26].

We must not forget that silk fibroin from silkworm is of great interest as a biomaterial with numerous applications in biomedicine including the development of films, membranes, gels, sponges, powders, and scaffolds [27]. Applications include burn-wound dressings, enzyme immobilization matrices, nets, vascular prostheses, and structural implants [27], including nerve grafts in neuroscience [28]. In recent years, with an improved understanding of the fundamental structures and properties of silk, along with options to improve the purification of the native fiber structural core (fibroin) without residual contaminating proteins (e.g., sericin), degradable silk biomaterials have been generated which are also biocompatible and show acceptable rates of biodegradability [29]. In this context, it is not surprising that there are numerous studies analyzing the in vitro enzymatic degradation of fibroin and raw silk. Indeed, silk fibroin degrades in vitro and in vivo in response to different proteolytic enzymes including actinase (a *Streptomyces* sp., enzymatic mix degrading actin) [30], α-chymotrypsin, collagenase, papain [27,29], protease XIV, and protease XXI (both proteases are really a mix of proteolytic enzymes isolated from *Streptomyces* species including at least 3 different proteases, of which at least one is an extracellular serine protease) [16].

In our case, and in order to identify some of the putative enzymes involved in raw silk biodegradation, we discarded a classical approach consisting of cell fractionation and successive purification steps using classical chromatographic techniques due to their laborious and time-consuming nature. Instead, we decided to adopt a strategy that takes advantage of the enormous potential of genome sequencing techniques and genome analysis. Thus, sequencing of the *B. licheniformis* genome and analysis of the different proteases and peptidases encoded in it allowed us to detect a total of 135 genes encoding enzymes with possible proteolytic activity (proteases and peptidases). This analysis combined with a prediction of their putative extracellular location (extracellular enzymes) by in silico analysis allowed us to conclude that a total of 20 proteases/peptidases could be considered as secreted enzymes with an extracellular location. This group should include the best candidates for enzymes involved in silk biodegradation, a process which, based on the different microscopic observations made and the structure of silk itself, should be extracellular. In fact, some evidence indicates that enzymatic degradation of silk and other biomaterials is a two-step process. The first step is adsorption of the enzyme onto the surface of the substrate through surface-binding domains, and the second step is hydrolysis of the peptide bonds [30]. 

Analysis of these 20 extracellular proteases/peptidases allowed us to discard some of them according to their biological function (e.g., those enzymes of the carboxypeptidase type that are involved in wall peptidoglycan processing). Among the remaining enzymes, subtilisin Carlsberg (a Ser-endopeptidase) particularly attracted our attention because it is an extracellular alkaline serine protease, catalyzing hydrolysis of proteins and peptide amides, that shows high specificity for aromatic and hydrophobic amino acids at the P1 position of the substrate [19,20]. This enzyme was, therefore, an ideal candidate for extensive biodegradation of raw silk, as both sericin and fibroin would hypothetically contain numerous potential cleavage sites. First analyses with commercial subtilisin from *B. licheniformis* confirmed this fact: incubation of raw silk with the enzyme produced both a significant release of amino acids into the reaction medium, and also a visually evident degradation of the silk ball in the reaction tubes. Homologous overexpression of the *las*B gene encoding subtilisin Carlsberg in the strain from which it was isolated (strain IIVV-SD3) confirmed its key role in silk biodegradation, as the transformants tested showed higher degradation efficiency compared to the wild-type strain. Although in vitro biodegradation data with commercial subtilisin confirmed its predominant role in silk biodegradation, we cannot rule out that other proteases could be involved in the process. Among the best candidates we should mention the two bacillopeptidases F detected (proteins 2 and 3 in Table S1—Supplementary Material). Bacillopeptidases F are extracellular fibrinolytic serine proteases [31] belonging to the superfamily of subtilisin-like serine proteases. These enzymes are expressed at the beginning of the stationary phase in *B. subtilis*. Inactivation of their encoding genes does not affect either vegetative growth or sporulation, and they probably function as scavenging enzymes to supply the cell with amino acids derived from protein degradation [32].

In a similar approach, analysis of the *P. aeruginosa* IIVV-SD1 strain sequenced genome allowed us to detect 9 extracellular proteases/peptidases. Among all of them, our attention was primarily focused on elastase, or LasB protein, for several reasons. First, because LasB is a protease with broad specificity, although it favors hydrophobic or aromatic amino acid residues, with preference to Phe and Leu at the P1′ and Gly in P1 positions [22]. Since Gly is higly abundant, and to a lesser extent Leu and Phe, in pine processionary fibroin [11], we could assume the existence of numerous cleavage sites for this protease. Second, *P. aeruginosa* elastase, or pseudolysin, is the predominant protease in the *P. aeruginosa* secretome and is an important virulence factor [23]. Finally, porcine elastase was commercially available for performing some preliminary in vitro degradation tests, although as we have already indicated above its cleavage specificity is different to that of *P. aeruginosa* elastase. These tests confirmed that porcine elastase was able to efficiently degrade raw silk. However, as there might be some differences in specificity, or degradation efficiency, between porcine and *P. aeruginosa* elastase, the latter was efficiently expressed in *E. coli*. The recombinant protein obtained was able to degrade crude silk in vitro, as evidenced by its degradation into L-Leucine equivalent released into the reaction medium. 

Confirmation that *P. aeruginosa* elastase could efficiently degrade silk may have important biosanitary implications. *P. aeruginosa* is an opportunistic bacterial pathogen present in many environments which can cause fatal and debilitating disease, especially in patients whose immune responses are compromised, and who are unable to clear an initial infection [23]. In fact, *P. aeruginosa* can adapt to many situations, often adopting a biofilm mode of growth within which the organism is able to survive antibiotic challenges [33]. Since silkworm silk is the starting material for the production of numerous materials for medical use in patients (sutures, films, membranes, gels, sponges, powders, and scaffolds, among others), as we mentioned above, potential colonization of *P. aeruginos*a of these materials and their putative degradation by elastase should not be underestimated and could be the cause of numerous problems in patients. 

The finding that enzymes such as subtilisin and elastase can efficiently degrade pine processionary silk, and possibly also silkworm silk (to our knowledge their ability to degrade silkworm silk has not been described so far) opens interesting perspectives on this topic and expands the range of enzymes and microorganisms involved in the biodegradation of silk materials.

Finally, we would like to emphasize some other practical applications that could be derived from the findings described in this manuscript. First, we should mention that current management of pine processionary moths includes a combination of preventive techniques, such as planting policies and methods for early detection, curative methods such as trapping of adults and larvae, elimination of winter nests, application of insecticides and bioinsecticides, and pheromone treatment, especially in small urban areas [4]. Unfortunately, these methods may provide insufficient levels of control or endanger the health of human and domestic animals, particularly in urban parks and recreational suburban areas. Moreover, insecticide applications in the anthropized sites may be ineffective, as some parts of the plants remain untreated and spraying in inhabited areas often triggers complaints from residents. Biological control has to be stringently applied to achieve a satisfactory level of management [4]. However, in big forests, spraying with synthetic pesticides, biopesticides such as Spinosad [34], Bt protein [35], or biocontrol agents like *Bacillus thuringiensis* or entomopathogenic fungi [36,37] is the only method yielding some effectiveness [34]. Given the high protection to environmental conditions that silk nests provide to caterpillars, their disorganization or biodegradation by the application of strains of subtilisin- or elastase-producing microorganisms could contribute to increasing the efficacy of other treatments. Our findings especially support the potential use of bioinsecticide-producing bacterial strains with the ability to synthesize elastase or subtilisin to control pine processionary pest. Unfortunately, and to the best of our knowledge neither *B. licheniformis* nor *P. aeruginosa* have known bioinsecticidal activity. Moreover, the use of the latter microorganism as a BCA would not be possible given its character as an important opportunistic pathogen for humans and other animals [22]. However, it is interesting to mention that some strains of *B. thuringiensis* include genes encoding for subtilisins in their genome [38]. In these strains subtilisin is a virulence factor that could be involved in efficient larval body utilization during the infection process [38]. It has also been reported that proteases, including subtilisin, play a significant role in the conversion of δ-protoxins to active toxins [39,40]. Putting together all these data we could hypothesize that the selection of *B. thuringiensis* strains with both good subtilisin and bioinsecticidal activities toward pine processionary caterpillars could be a more efficient treatment than biofumigation with Bt protein alone, since the larvicidal effect of the endotoxin would be added to the biodegradative effect on the silk. The inoculation by biofumigation of these kind of selected strains on silk nests could allow their active growth in the nests and thus favor a more active transmission by a higher rate of infection among caterpillars probably allowing an improved control of the pest.

Finally, as we have indicated above, in some countries the removal and subsequent burning of winter nests is a common management practice to reduce the incidence of the pest [4,18]. As we mentioned, the amino acids alanine, glycine and serine are particularly abundant in silk, and to a lesser extent other amino acids such as aspartic, glutamine, tyrosine and arginine are also present. All these amino acids have many different industrial applications and are obtained by chemical or biological methods (fermentations carried out by selected microorganisms) [41]. It is possible that biodegradation of pine processionary nests under controlled conditions in an industrial environment, especially by *B. licheniformis*, which has a greater biodegradation capacity than *P. aeruginosa,* could be an interesting alternative for the industrial production of some of these amino acids. The case of serine is particularly interesting since this amino acid is currently used in industries ranging from food to cosmetics, and demonstrates many novel potential applications in pharmaceutical industries [42]. Currently, L-serine production relies mainly on extraction from protein hydrolysates, chemical synthesis, or enzyme or cellular conversion from the precursor glycine plus a C1 compound such as methanol. However, dependence on high-priced substrates, low yield, and environmental pollution make current production methods less attractive and non-sustainable [42]. Perhaps production of serine from the biodegradation of a waste material, such as silk from pine processionary nests, could be an interesting alternative worth investigating, although is evident that some kind of exopeptidase should also be involved in the degradation of the peptides produced by the subtilisin activity in order to release serine and allow its accumulation into the culture supernatant.

In summary, we would like to highlight that the finding that silk from pine processionary, and possibly also silkworm silk, can be biodegraded by subtilisin and elastase type enzymes opens up a wide range of perspectives that deserve to be investigated, including their repercussions in biomedicine, pest control and possible applications as a renewable source for the industrial production of certain amino acids. 

## 4. Materials and Methods

### 4.1. Silk Processing

Silk was obtained from nests collected from a pine forest in the area known as La Candamia (42°34′54.6″ N 5°32′08.0″ W), close to the city of León (Spain). For the isolation of putative silk degrading microorganisms small silk samples of 1–2 g were taken in the field directly from nests with an evident state of degradation using sterile scissors and forceps and immediately introduced into sterile Falcon tubes to avoid any accidental contamination. The silk used for further microbial or enzymatic degradation studies in the laboratory was obtained from the collection of young nests that were placed in plastic bags, and autoclaved to kill any caterpillars. The silk was then manually cleaned using combs and tweezers in order to remove any remains of sticks, pine needles, caterpillars and caterpillar excrement naturally present in the nests. After careful cleaning, the silk was re-sterilized in an autoclave and stored in sterile Falcon tubes at 4 °C until use.

### 4.2. Acid Hydrolysis of Silk and Amino Acid Analysis by HPLC

Acid hydrolysis of raw silk was carried out following the methodology described by Hess and colleagues [43] with minor modifications. Briefly, 2 mg silk was suspended in 1 mL 6N HCl in a glass tube. Phenol (0.02%) was included as a protective agent to reduce the loss of some residues during hydrolysis [44]. Traces of oxygen were replaced by repeated flushing with nitrogen. The closed tube was heated at 110 °C for 24 h to achieve complete hydrolysis. Next, the liquid was evaporated in a rotary evaporator with a vacuum pump(Buchi R300, Barcelona, Spain) at 65 °C for 8 h. The residue was dissolved in water (1 mL) and evaporated to remove further HCl. The final residue was dissolved in 200 μL of MQ water and stored at −20 °C until use. The amino acid composition of the hydrolyzate was analyzed, previous *o*-phthalaldehyde derivatization, by reversed-phase HPLC (RP-HPLC) [45] on a Zorbax Eclipse XDB C18 (4.6 × 150 mm; 5 μm) column (Agilent Technologies, Saint Claire, CA, USA) using an Agilent 1200 Liquid Chromatograph equipped with a quaternary pump delivery system (Saint Claire, CA, USA) (G1311A), a preparative autosampler (G1329A), a diode array multi-wavelength detector (G7115A), a fluorescence detector (G1321A), and an analytical fraction collector (G1364F) equipped with an Autosampler Thermostat (G1330B). Samples of 2.5 μL were injected, and detection was carried out at 338 nm. Chromatographic conditions were the same as reported by Bartolomeo and Maisano [45]. Quantification of amino acids was carried out according to the area of their respective peaks by comparison with authentic amino acid peaks. Amino acid standard samples contained 20, 50, 130, 250, or 500 pmol/μL of an amino acid standard mixture together with 0.5 mM norvaline.

### 4.3. Isolation and Identification of Silk-Degrading Bacterial Strains

Twenty mg of silk collected from an old nest with evident symptoms of biodeterioration were deposited at the bottom of a 10 mL polypropylene sterile tube. Next, 2.0 mL of yeast extract (0.25%, *w*/*v*), and 0.1 mL of a salt mixture [ZnSO_4_ x 7H_2_O, 4 g/L; FeSO_4_ x 7H_2_O, 9 g/L; CuSO_4_ x 7H_2_O, 0.18 g/L; H_3_BO_3_, 0.026 g/L; (NH_4_)_3_Mo_4_O_7_ x 4H_2_O, 0.017 g/L, MnSO_4_ x 4H_2_O] were added and the mix was brought to a final volume of 5 mL with sterile water. The mix was incubated up to 60 days at RT. Once silk degradation was visually observed, two more enrichment rounds were carried out under the same conditions, except 0.1% glucose was added to accelerate bacterial growth, and in turn, the biodegradation process. Then tenfold dilutions were spread on the surface of Plate Count Agar (PCA) (Condalab, Torrejón de Ardoz, Spain) plates containing natamycin (200 µg/mL) to avoid fungal growth. Plates were incubated at 28 °C until bacterial colonies developed. Bacteria representative of different macro and microscopic morphological types were selected at random and conserved in Nutrient Agar plates (Condalab) at 4 °C until use. 

To test which of the bacterial isolates had the ability to biodegrade silk, pure cultures in the presence of sterile silk were developed as previously indicated, including 0.1% glucose. Cultures were inoculated with 500 µL of a pure bacterial culture (OD 1 at 600 nm). Positive biodegrading strains were selected for their ability to visually solubilize silk and for their ability to release ninhydrin positive products from the degraded silk. Amino acid release was quantified with ninhydrin as reported by Sun et al. [46] and the color produced was determined spectrophotometrically at 580 nm against a standard curve containing known amounts of leucine.

Silk degrading isolates were identified by 16S rRNA sequencing. Briefly, genomic DNA extraction was performed as described by Hopwood et al. [47]. Amplification of 16S rRNA genes was carried out using oligonucleotides 27F and 1492R [48]. Isolates were identified by comparison with the corresponding sequences of type strains found on Ez Taxon-e database [49] (http://www.ezbiocloud.net/eztaxon/identify accessed on 18 February 2022). Sequence alignment as well as phylogenetic trees were carried out using the MEGA 6 software [50].

### 4.4. Sequencing and Genome Assembly of Silk Degrading Strains

Genomic DNA was sent to Macrogen (Seoul, South Korea) for Illumina sequencing. Illumina TruSeq Nano DNA Kit was used to generate the Illumina library according to the manufacturer’s specifications. Illumina sequencing was performed on a Novaseq-6000 producing paired-end 2 × 150 bp reads. Raw Illumina reads were analyzed with FASTQC v0.11.8 (http://www.bioinformatics.babraham.ac.uk/projects/fastqc accessed on 18 February 2022) to obtain quality statistics, then Trimmomatic v0.38 [51] was used to remove adapter sequences and trim out bases of low quality (minimum base quality 35 and minimum read length 35 bp). The Illumina reads were then aligned against reference genomes (*B. licheniformis* GCF_002074095.1 and *P. aeruginosa* GCF_000006765.1) with BWA v0.7.17 (http://bio-bwa.sourceforge.net/ accessed on 18 February 2022). During mapping, duplicate reads were removed using Sambamba v0.6.8 (http://lomereiter.github.io/sambamba/ accessed on 18 February 2022). After removing duplicates and identifying variants with SAMTools (http://samtools.sourceforge.net/ accessed on 18 February 2022) information about each variant was gathered and classified by chromosomes or scaffolds. In order to discover annotation information, such as amino acid changes by variants, SnpEff v4.3t (http://snpeff.sourceforge.net/ accessed on 18 February 2022) was used.

Genomes of the *B. licheniformis* and *P. aeruginosa* strains were assembled using SPAdes v. 3.15.4 [52]. Quality of the results was analyzed using Quast v. 5.2.0 [53], comparing it to the reference genome of each species to check its coverage. The assembled genomes were processed with BUSCO v5.3.25 [54] to make a preliminary annotation and predict the proteins with Prodigal [55]. Subsequently, the predicted proteins were annotated with Blastp v2.9.0-2 [56] against the NCBI SwissProt database [57]. Finally, using an R v4.2.1 [58] script, a functional annotation was obtained by propagating the annotation to obtain the GO terms [59] and the InterPro [60] protein families corresponding to each annotated protein. 

Both whole genome shotgun projects were deposited at DDBJ/ENA/GenBank as a Bioproject (ID PRJNA894474) that includes the *B. licheniformis* (IIVV-SD3 strain) genome project (accession number JAPDGT000000000) and the *P. aeruginosa* (IIVV-SD1 strain) genome project (accession number JAPDGU000000000).

### 4.5. Silk Biodegradation in Liquid Media

Analysis of silk degradation in liquid media was carried out in triplicate in 50 mL Falcon tubes with a perforated plug and a valve for allowing gas exchange. Tubes contained 10–20 mg of sterile silk suspended in 30 mL of minimal medium [K_2_HPO_4_, 40 mM; KH_2_PO_4_, 22 mM; MgSO_4_ x 7H_2_O, 0.8 mM; KNO_3_, 10 mM; glucose, 0.5% (*w*/*v*) and 30 µL of a salt mixture as indicated above in Section 4.3]. Tubes were inoculated with 10^6^ bacterial cells/mL and incubated at 30 °C. Samples (500 μL) were removed at different times and silk degradation quantified by measurement of free amino acids in the culture supernatant by performing a ninhydrin test [46]. Amino acid quantification was carried out at 570 nm by comparison with a standard curve of leucine. 

### 4.6. Scanning Electron Microscopy (SEM) and Transmission Electron Microscopy (TEM)

Raw silk samples were processed according the Bertazzo protocol [61]. Briefly, silk samples were fixed with electron microscopy grade glutaraldehyde (TAAB Laboratories, Berks, UK) at a final concentration of 2.5% in phosphate buffered saline (PBS) for 2 h at 4 °C. Samples were washed three times in PBS. Next, they were refixed in the dark at RT with 2% osmium tetroxide (TAAB Laboratories) in PBS for 2 h. Finally, three 30-min washes were performed with PBS. Next, samples were dehydrated by immersion in solutions of increasing ethanol concentration for 30 min each step (30%, 50%, 70%, 90%, 3 × 96% and 3 × 100%). Sample drying was carried out using a critical-point desiccator model CPD 030 from BAL-TEC Inc (Liechtenstein). Samples were immediately gold-coated and mounted on aluminum stabs to be examined with a Scanning Electron Microscope model JSM-6480 LV from JEOL (Tokyo, Japan).

Samples for TEM analysis were prepared according to the protocol described by Wang et al. [28] and observed under a transmission electron microscope model JEM-1010 (JEOL).

### 4.7. Enzymatic Assays for In Vitro Silk Degradation

In vitro enzymatic degradation of silk was tested using commercial porcine pancreatic elastase (≥8 U/mg of protein) (Worthington Biochemical Corporation, Lakewood, NJ, USA) or commercial lyophilized subtilisin A (7–15 U/mg of protein) from *B. licheniformis* (Merck KGaA, Darmstadt, Germany). Reactions contained 2 mg clean, sterile silk and different amounts of enzyme and were carried out under the assay conditions indicated by the supplier.

### 4.8. DNA Isolation and Manipulation

Small-scale total DNA from *B licheniformis* and *P. aeruginosa* was extracted using Illustra^TM^ Bacteria genomicPrep Mini Spin Kit (Illustra, GE Healthcare Bio-Sciences AB, Uppsala, Sweden). Large-scale total DNA isolation was carried out as described by Hopwood et al. [47]. Small scale plasmid DNA isolation from *E. coli* was carried out using the boiling method [62] or alternatively using Illustra^TM^ plasmidPrep Mini Spin Kit (Illustra). DNA manipulations were performed according to standard procedures [63].

### 4.9. Heterologous Expression in E. coli of a P. aeruginosa lasB Elastase-Encoding Gene

A *P. aeruginosa* LasB-encoding gene was amplified from genomic DNA by PCR using F-LasB (5′-ATGAAGAAGGTTTCTACGCTTGACC-3′) and R-LasB (5′-TTACAACGCGCTCGGGCA-3′) primers and Phusion Hot Star II DNA polymerase (Thermo Fisher Scienfic, Waltham, MA, USA). The PCR fragment was ligated to Champion^TM^ pET SUMO expression vector (Thermo Fisher Scientific) and the ligation reaction was transformed into *E. coli* One Shot^®^ Mach1TM (Thermo Fisher Scientific) competent cells following the manufacturer’s instructions. The resultant recombinant plasmid (pET SUMO-LasB) was sequenced to confirm that no nucleotide changes had occurred during PCR amplification, and the insert had been introduced in the right orientation. 

Next, this recombinant plasmid was introduced into competent cells of *E. coli* BL21(DE3) One Shot strain (Thermo Fisher Scientific) to check putative LasB overexpression. A selected recombinant clone was grown in an orbital shaker (37 °C/220 r.p.m) in LB liquid medium (100 mL) containing 50 μg/mL of kanamycin until an O.D. 600 nm of 0.5 was reached. Overexpression was induced by adding 0.75 mM IPTG and incubating at 37 °C for an additional 6 h. Cells were recovered by centrifugation and pellet resuspended in Buffer A (NaH_2_PO_4_, 100 mM; Tris-HCl, 10 mM; guanidine hydrochloride, 6M; NaCl, 0.5 M; 2-mercaptoethanol, 1 mM; pH 8.0). Cells were disrupted by sonication in an ice bath using 15 cycles of 3–4 s bursts (90–100 W) with a Branson Sonifier B-12 (Thermo Fisher Scientific). Inclusion bodies were solubilized at RT for 4 h in a rotating arm. Cellular debris were removed by centrifugation (4 °C/15,000× *g*/10 min). 

Recombinant protein was purified onto 0.5 mL of HisPure^TM^ Ni-NTA resin (Thermo Fisher Scientific), previously equilibrated by extensive washing in Buffer A, by overnight incubation at 4 °C in a rotating arm. Contaminant and unbound proteins were removed by washing (3 times) with 10 vol Buffer A containing 20 mM imidazole. Next the resin was packed into a 1 mL small column and the recombinant protein was refolded and immobilized on the Ni-NTA matrix using step-by-step removal of guanidine hydrochloride using a Refolding Buffer (Tris-HCl, 50 mm; NaCl, 100 mM; glycerol, 10% (*w*/*v*); pH 7.0) containing decreasing concentrations (5M, 4M, 3M, 2M, 1M, 0.5M, 0.25M) of guanidine hydrochloride. All the refolding steps were done at 4 °C by slowly adding 2 mL of each Refolding Buffer to the column. Finally, the protein bound to the Ni-NTA matrix was conserved in Refolding Buffer at 4 °C until use. A small amount of resin (10–20 μL) was analyzed by SDS-PAGE to check the purified refolded protein. Protein concentration was visually estimated by comparison with known amounts of BSA run in an SDS-PAGE gel in parallel.

### 4.10. Analysis of Silk Degradation by Recombinant Elastase

The ability of recombinant LasB-SUMO protein bond to Ni-NTA resin to degrade silk was tested in in vitro reactions containing 10 μg recombinant protein and 2 mg silk in a total volume of 1.5 mL Tris-HCl, 100 mM (pH 8.0) buffer. Reactions were incubated at 37 °C up to 12 h in a rotating arm. Samples of 100–200 μL were removed at different times and silk degradation was estimated by release of amino acids into the solution using the ninhydrin assay.

### 4.11. Homologous Expression in B. licheniformis of a Subtilisin Carlsberg-Encoding Gene (subC)

A recombinant plasmid pHY300PLK-pSubC was obtained by cloning the subtilisin Carlsberg-encoding gene (*sub*C) located in the genome of *B. licheniformis* IIVV-SD3 into the shuttle vector pHY300PLK [64]. The gene and its promoter region were PCR-amplified from genomic DNA using F_PSubC (5′-AATCAGGATCCCCGTTTCTGTATGCGATA-3′) and R_PSubC (5′-CTTATCCCGGGTTATTGAGCGGCAGCTTCGAC) primers and high affinity Phusion Hot Star II DNA polymerase (Thermo Fisher Scienfic). A PCR product of the expected size was purified from an agarose gel using the Freeze-Squeeze method [65] and later digested with *Bam*HI and *Xba*I restriction enzymes in order to generate compatible cohesive ends that allowed its cloning in the *Bam*HI-*Xba*I digested pHY300PLK vector. Ligation mix was transformed into *E. coli* ET12567 strain competent cells following standard protocols [63]. A clone containing the recombinant plasmid pHY300PLK-pSubC was selected by restriction analysis and sequenced to confirm the absence of undesired mutations. Next the recombinant pHY300PLK-pSubC plasmid was introduced into *B. licheniformis* IIVV-SD3 by electroporation following the protocols described by Xue et al. [66]. DNA from transformants was isolated with GeneJET Plasmid Miniprep Kit (Thermo Fisher Scientific) and the presence of recombinant plasmid was checked by PCR using pHY300PLK_F (5′-GTCAGATTTCGTGATGCTTGTC-3′) and pHY300PLK_R (5′-GGATCAACTTTGGGAGAGAGTTC-3′) primers. These primers anneal to both sides of the multicloning site (MCS) of the pHY300PLK vector and allowed amplification of the whole insert cloned in pHY300PLK-pSubC plasmid, whereas no amplification was observed from total DNA from *B. licheniformis* IIVV-SD3·used as negative control. Additional verification was carried out by restriction analysis of plasmid DNA isolated from the transformants. Two transformants were selected for further studies and copy number of *sub*C gene determined by quantitative PCR (qPCR). Total DNA from both transformants and IIVV-SD strain was isolated using Illustra™ Bacteria genomicPrep Mini Spin Kit (Illustra).

For plasmid copy number determination in the transformants, two primer sets specific to *sub*C and *rpo*B genes, this last encoding RNA polymerase β-subunit, were designed. The *sub*C gene was present as a single copy in pHY300PLK-pSubC plasmid and also in the chromosomal DNA, and likewise the *rpo*B gene has only one copy in the bacterial genome. Primer sets were designed using Primer3 software [67]. A 100 bp internal fragment of *sub*C gene was amplified using F_subC-qPCR (5′-TTCACAAGTCCGCAACCGTC-3′) and R_subC-qPCR (5′-TTATTGAGCGGCAGCTTCGAC-3′) primers. In a similar way a 70 bp internal fragment of *rpo*B gene was amplified using F_rpoB-qPCR (5′-ACCTCTTCTTATCAGTGGTTTCTTGAT-3′) and R_rpoB-qPCR (5′-CCTCAATTGGCGATATGTCTTG-3′) primers. Internal fragments of both genes were amplified by conventional PCR. The reaction mixture of 50 µL contained 0.1 mM dNTP, 0.5 µM of each primer, 0.05 U Paq5000 DNA polymerase and 25 ng of *B. licheniformis* genomic DNA as a template. PCR amplification was performed with a Mastercycler Gradient thermocycler (Eppendorf, Hamburg, Germany), according to the following program: an initial denaturation at 98 °C for 3 min, followed by 30 cycles of 30 s at 98 °C, 30 s at 60 °C and 1 min at 72 °C; a final extension at 72 °C for 10 min. Both PCR products were purified using the NucleoSpin Gel and PCR Clean-up kit (Macherey-Nagel GmbH & Co. KG, Duren, Germany) and quantified on NanoDrop 2000 (Thermo Fisher Scientific). Since the size and nucleotide composition of each amplicon are known, the number of copies of each PCR amplified fragment was easily estimated using an online calculator (http://cels.uri.edu/gsc/cndna.html accessed on 3 October 2022). Then, PCR products were serially 10-fold diluted and used as templates to generate qPCR standard curves, one for each gene (Supplementary material Figure S2A).

The *B. licheniformis* IIVV-SD3 wild type and two *B. licheniformis* IIVV-SD3 transformants harboring pHY300PLK-pSubC plasmid were cultured in flasks containing 50 mL of LB (supplemented with 30 μg/mL tetracycline for culture transformants). Total DNA was extracted from both cultures when they reached approximately half the exponential phase (OD_600nm_ 0.8). DNA extraction was performed using the Illustra^TM^ bacteria genomicPrep Mini Spin Kit (GE Healthcare), following manufacturer’s instructions. The concentration of extracted DNA was measured using NanoDrop 2000 (Thermo Fisher Scientific). Genomic DNA was serially diluted 10-fold and copy number of each gene was estimated using the previous standard curves (Supplementary material Figure S2B).

Real-time QPCR *SYBR Green* amplification and analyses were performed using a Mx3005P QPCR system and software (Agilent technologies). The real time qPCR mixture of 20 µL contained 1X SYBR Green master mix (Takara, Shiga, Japan), 0.2 μMol of each primer and 5 µL bacterial DNA. Nuclease-free water was used to bring the reaction volume to 20 μL. The thermal cycling protocol was as follows: initial denaturation for 2 min at 95 °C followed by 40 cycles of 30 s at 95 °C and 30 s at 60 °C. After amplification, a melting curve analysis with a temperature gradient of 0.1 °C/s from 55 to 95 °C was performed to confirm that only specific products were amplified. All analyses were performed as three independent experiments with three replicates for each dilution.

## 5. Conclusions

Our current study extends the knowledge on the molecular mechanisms involved in the microbial degradation of silk materials and fibroin-like proteins. For the first time we demonstrate that bacteria such as *P. aeruginosa* and *B. licheniformis* can efficiently biodegrade silk materials, and more specifically silk produced by the pine processionary. For this purpose, these microorganisms use common enzymes in the bacterial world such as elastase produced by *P. aeruginosa* or subtilisin produced by *B. licheniformis*. Therefore, any microorganism producing these types of enzymes would be susceptible to degrade silk materials.

## Data Availability

Not applicable.

## Supplementary Materials

The following supporting information can be downloaded at: https://www.mdpi.com/article/10.3390/ijms232315253/s1.
