# Supplementary material for: Biodegradation of Pine Processionary Caterpillar Silk Is Mediated by Elastase- and Subtilisin-like Proteases
Source: Int J Mol Sci. 2022 Dec 3;23(23):15253. doi: 10.3390/ijms232315253 (PMC9741414; doi:10.3390/ijms232315253)
Supplement: Supplementary file 1 [file ijms-23-15253-s001.zip › Figure S1.pdf]

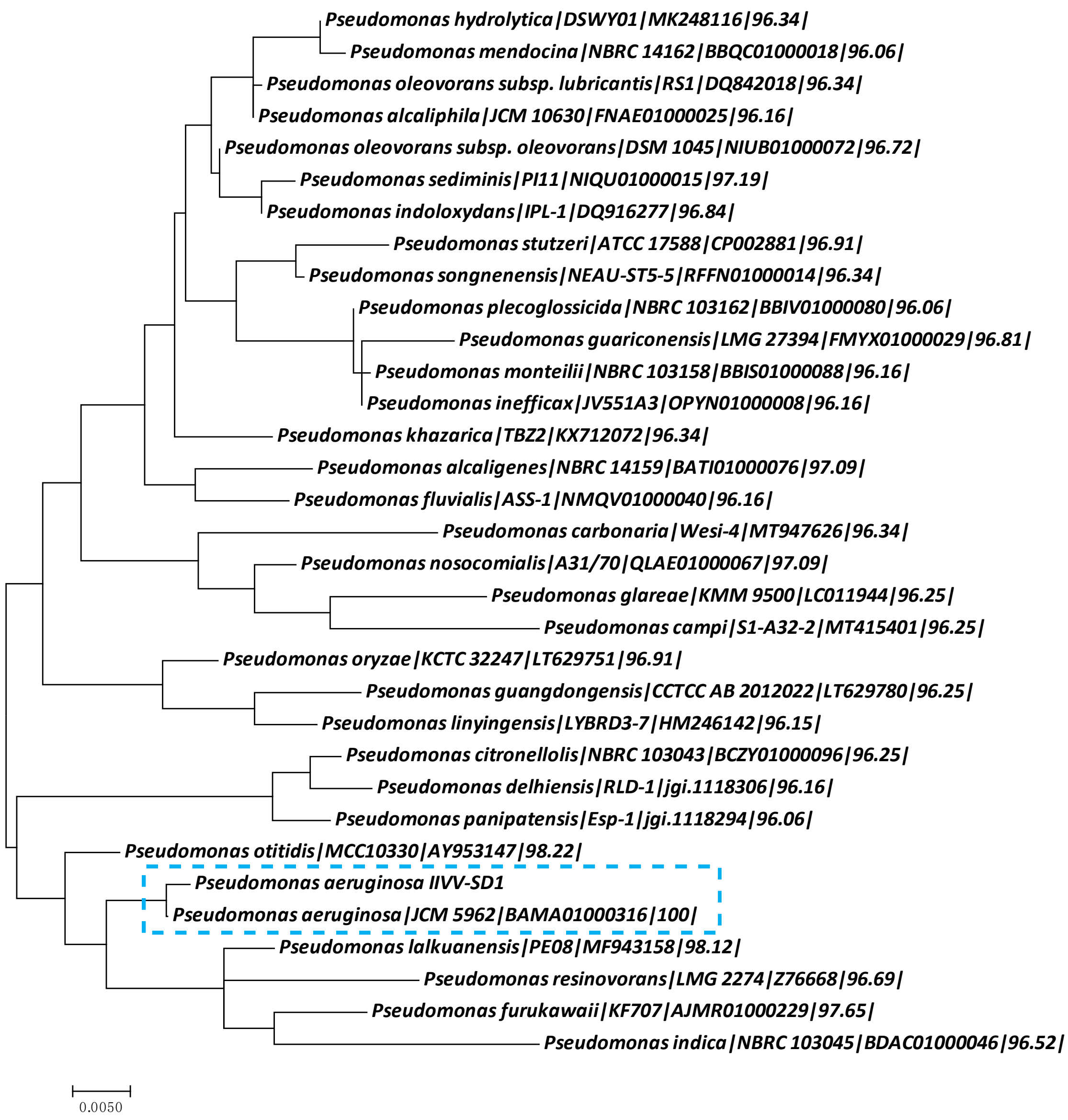

Figure S1A. Phylogenetic tree based on 16S rDNA sequences phylogenetically closer to *Pseudomonas aeruginosa* IIVV-SD1. Molecular Phylogenetic analysis was inferred by using Maximum Likelihood method. The tree with the highest log likelihood (-5040,15) is shown. Initial tree for the heuristic search were obtained automatically by applying Neighbor-Join and BioNJ algorithms to a matrix of pairwise distances estimated using the Maximum Composite Likelihood approach, and then selecting the topology with superior log likelihood value. The tree is drawn to scale, with branch lengths measured in the number of substitutions per site. The analysis involved 33 nucleotide sequences. All positions containing gaps and missing data were eliminated. There were a total of 1384 positions in the final dataset. Evolutionary analyses were conducted in MEGA6 **[50].**

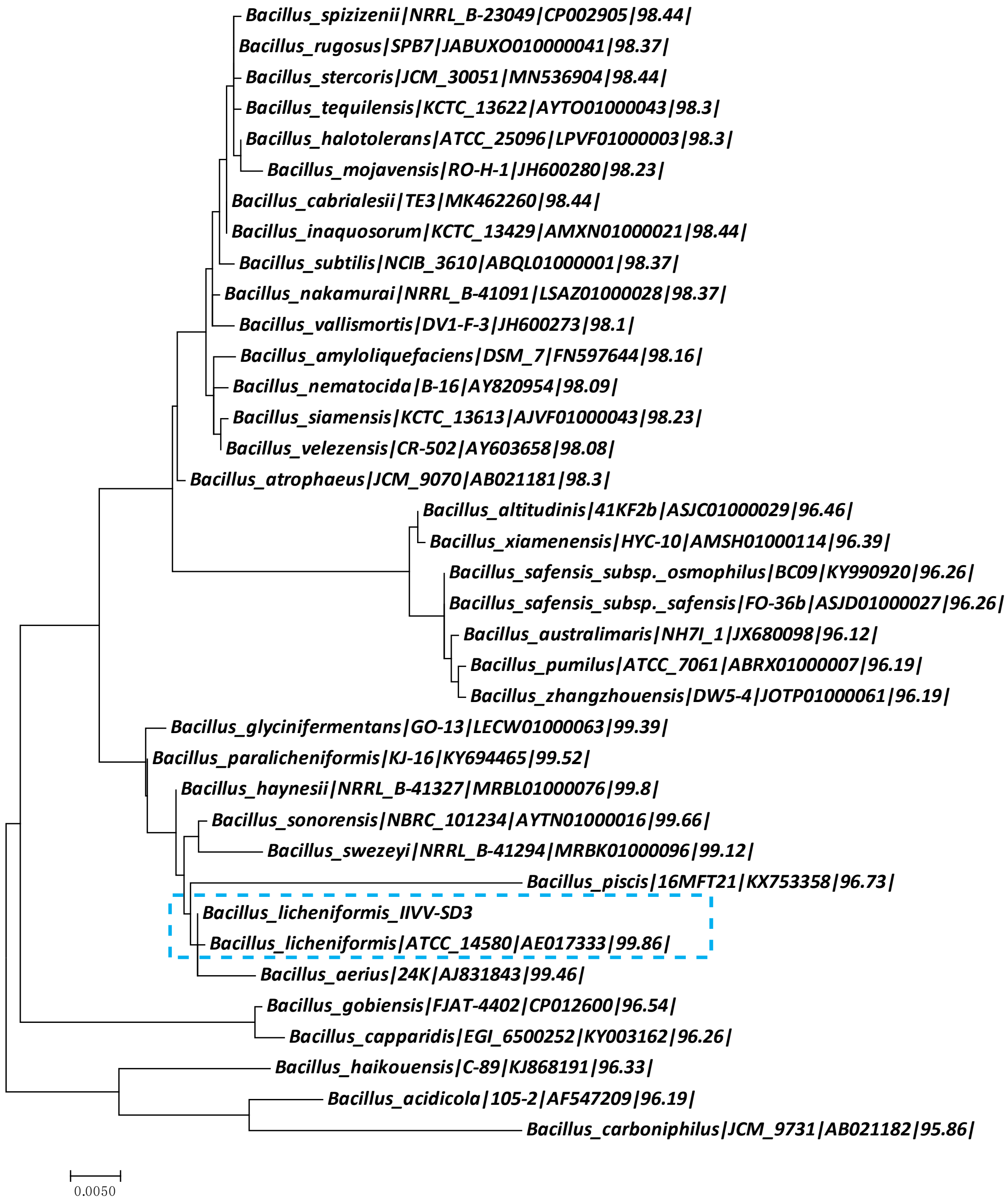

Figure S1B. Phylogenetic tree based on 16S rDNA sequences phylogenetically closer to *Bacillus licheniformis* IIVV-SD3. Molecular Phylogenetic analysis was inferred by using Maximum Likelihood method. The tree with the highest log likelihood (-3983.38) is shown. Initial tree for the heuristic search were obtained automatically by applying Neighbor-Join and BioNJ algorithms to a matrix of pairwise distances estimated using the Maximum Composite Likelihood approach, and then selecting the topology with superior log likelihood value. The tree is drawn to scale, with branch lengths measured in the number of substitutions per site. The analysis involved 37 nucleotide sequences. All positions containing gaps and missing data were eliminated. There were a total of 1396 positions in the final dataset. Evolutionary analyses were conducted in MEGA6 **[50].**
