# Supplementary material for: Biodegradation of Pine Processionary Caterpillar Silk Is Mediated by Elastase- and Subtilisin-like Proteases
Source: Int J Mol Sci. 2022 Dec 3;23(23):15253. doi: 10.3390/ijms232315253 (PMC9741414; doi:10.3390/ijms232315253)
Supplement: Supplementary file 1 [file ijms-23-15253-s001.zip › Figure S2.pdf]

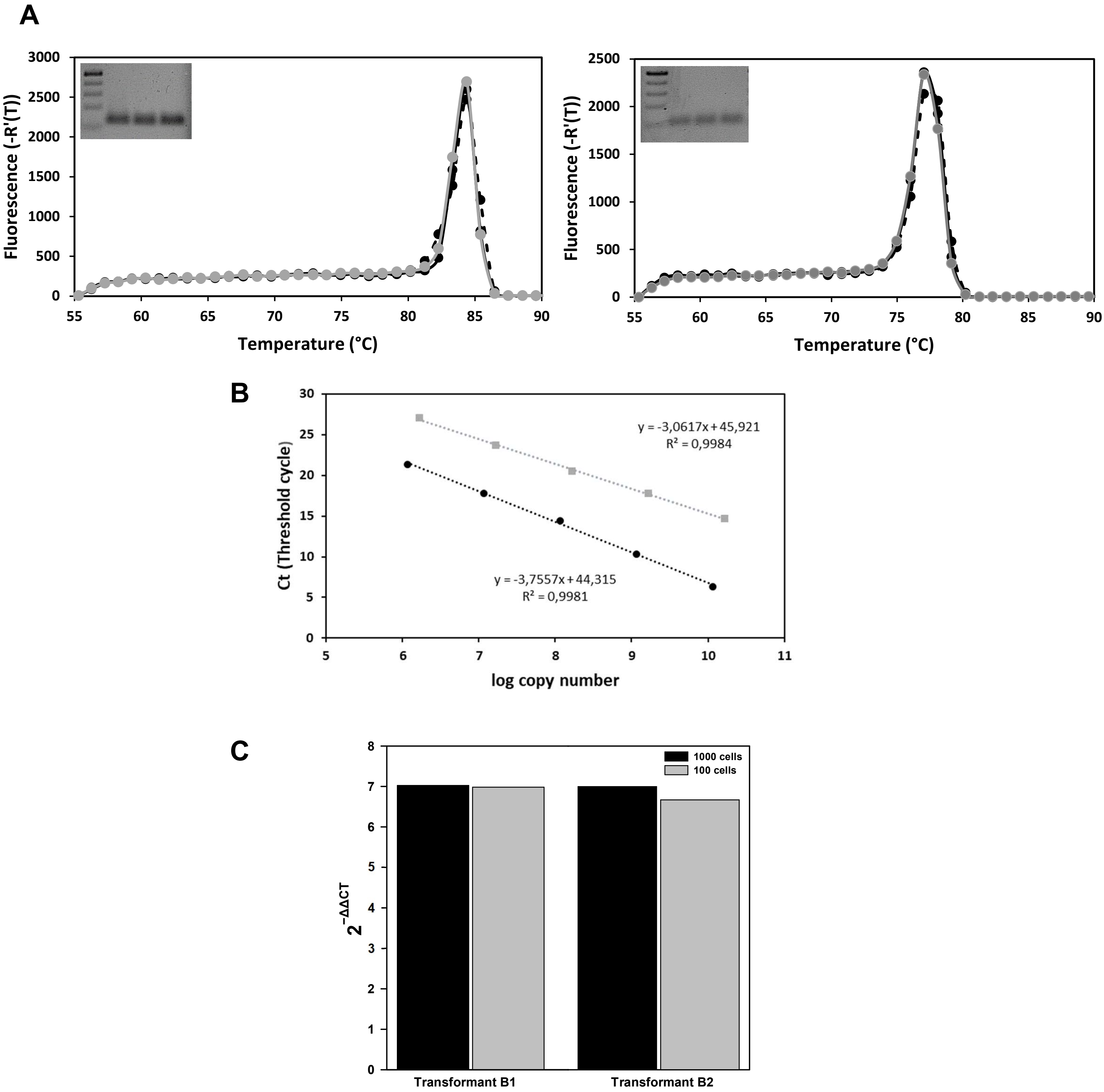


**Figure S2. Determination of *sub*C gene copy number in *B. licheniformis* IIVV-SD3 B1 and B2 transformants carrying the recombinant plasmid pHY300PLK-subC. (A).** Confirmation of the specificity of gene amplification of the *sub*C (left) and *rpo*B (right) genes by Real-Time PCR. Melting peaks were examined for the *sub*C and the *rpo*B products amplified with a quantitative standard PCR fragment sample (dashed line), total DNA from *B. licheniformis* WT (solid black line) and total DNA from *B. licheniformis* pHY300PLK-*sub*C transformant (solid gray line). Gel electrophoresis of the PCR products was performed on 1% agarose gel. Melting temperatures and amplicon sizes were 84°C and 70 bp for s*ub*C, and 77.5°C and 100 bp for *rpo*B genes, respectively. Identity of the amplified products was confirmed by DNA sequencing analysis. (**B**). qPCR standard curves for *sub*C (black) and *rpo*B (gray) gene quantification. (**C**). s*ub*C gene copy number normalized to that of the single copy gene *rpo*B in the B1 and B2 transformants as determined by the $2^{-\Delta\Delta CT}$ calculation
