# Supplementary material for: Biodegradation of Pine Processionary Caterpillar Silk Is Mediated by Elastase- and Subtilisin-like Proteases
Source: Int J Mol Sci. 2022 Dec 3;23(23):15253. doi: 10.3390/ijms232315253 (PMC9741414; doi:10.3390/ijms232315253)
Supplement: Supplementary file 1 [file ijms-23-15253-s001.zip › Table S1-R1.pdf]

Supplementary Material. Table S1. List of proteases/peptidases deduced from the annotated genome of *B. licheniformis* IIVV3 sorted by their putative cellular location determined by *in silico* analysis and according to their homology with proteins present in databases (NCBI id). (EXT: extracellular; CW: cell wall)

Number	Protein	Annotation	Cellular location	NCBI id
1	NODE_1_length_1554326_cov_186.887576_25	Glutathione hydrolase proenzyme (peptidase)	EXT	P54422.1
2	NODE_1_length_1554326_cov_186.887576_436	Bacillopeptidase F (Ser-endopeptidase)	EXT	P16397.2
3	NODE_1_length_1554326_cov_186.887576_437	Bacillopeptidase F (Ser- endopeptidase)	EXT	P16397.2
4	NODE_1_length_1554326_cov_186.887576_788	Minor extracellular protease Epr (Ser-endopeptidase)	EXT	P16396.1
5	NODE_1_length_1554326_cov_186.887576_1020	Ser-type D-Alal-D-Ala carboxypeptidase DacC	EXT	P39844.1
6	NODE_1_length_1554326_cov_186.887576_1387	Ser type D-alanyl-D-alanine carboxypeptidase DacF	EXT	P38422.2
7	NODE_1_length_1554326_cov_186.887576_1542	Uncharacterized protein YqgT (metallopeptidase)	EXT	P54497.1
8	NODE_2_length_1039096_cov_242.662227_247	Aminopeptidase YwaD (metallopeptidase)	EXT	P25152.2
9	NODE_2_length_1039096_cov_242.662227_302	Minor extracellular protease vpr (Ser-endopeptidase)	EXT	P29141.1
10	NODE_2_length_1039096_cov_242.662227_483	Gamma-DL-glutamyl hydrolase (Cys-type peptidase)	EXT	P96740.2
11	NODE_2_length_1039096_cov_242.662227_552	Peptidoglycan DL-endopeptidase CwlO (Cys-type peptidase)	EXT	P40767.2
12	NODE_3_length_407695_cov_224.282661_74	Probable peptidoglycan endopeptidase LytE (Cys-type peptidase)	EXT	P54421.1
13	NODE_3_length_407695_cov_224.282661_181	Subtilisin Carlsberg (Ser-endopeptidase)	EXT	P00780.2
14	NODE_3_length_407695_cov_224.282661_195	Minor extracellular protease Epr (Ser-endopeptidase)	EXT	P16396.1
15	NODE_4_length_387490_cov_255.809181_217	Peptidoglycan DL-endopeptidase CwlO (Cys-type peptidase)	EXT	Q65NQ9.1
16	NODE_4_length_387490_cov_255.809181_224	Glutamyl endopeptidase (Ser- endopeptidase)	EXT	P80057.2
17	NODE_6_length_190288_cov_236.934286_18	Carboxypeptidase 2 (metallocarboxypeptidase)	EXT	C5FYJ7.1
18	NODE_7_length_162504_cov_206.833098_93	Cell wall-associated protease (Ser-endopeptidase)	EXT	P54423.2
19	NODE_7_length_162504_cov_206.833098_122	Extracellular serine protease (Ser-endopeptidase)	EXT	P09489.1
20	NODE_13_length_20122_cov_320.430308_3	Ser-type D-alanyl-D-alanine carboxypeptidase DacA	EXT	P08750.3
21	NODE_1_length_1554326_cov_186.887576_60	Cys-type Gamma-D-glutamyl-L-lysine dipeptidyl-peptidase	CW	O35010.2
22	NODE_1_length_1554326_cov_186.887576_582	Uncharacterized zinc protease YmxG (Metalloendopeptidase)	CW	Q04805.3
23	NODE_1_length_1554326_cov_186.887576_1157	D-gamma-glutamyl-meso-diaminopimelic acid (Cys-type peptidase)	CW	O31852.1
24	NODE_1_length_1554326_cov_186.887576_1482	SpoIVB peptidase (Ser-peptidase)	CW	P17896.2
25	NODE_1_length_1554326_cov_186.887576_1708	Probable endopeptidase YddH (Cys-type peptidase)	CW	P96645.1
26	NODE_2_length_1039096_cov_242.662227_892	L-Ala--D-Glu endopeptidase (metalloendopeptidase)	CW	O32130.2
