# Supplementary material for: Biodegradation of Pine Processionary Caterpillar Silk Is Mediated by Elastase- and Subtilisin-like Proteases
Source: Int J Mol Sci. 2022 Dec 3;23(23):15253. doi: 10.3390/ijms232315253 (PMC9741414; doi:10.3390/ijms232315253)
Supplement: Supplementary file 1 [file ijms-23-15253-s001.zip › Table S2.pdf]

Supplementary Material. Table S2. Nucleotide and amino acid sequences of subtilisin Carlsberg (SubC) protein from *B. licheniformis* IIVV-SD3 and elastase (LasB) protein from *P. aeruginosa* IIVV-SD1 strains involved in the biodegradation of pine processionary raw silk. All the sequences can be found at DDBJ/ENA/GenBank (Bioproject ID PRJNA894474) that includes the *B. licheniformis* genome project (accession number JAPDGT000000000) and the *P. aeruginosa* genome project (accession number JAPDGU000000000)

Bacterial strain	Gene nucleotide sequence	Protein amino acid sequence
B. licheniformis IIVV-SD3 *sub*C gene and Subc protein	atgatgaggaaaaagagtttttggcttgggatgctgacggccttaatgctcgtgttcacg<br>atggcattcagcgattccgcttctgctgctcaaccggcgaaaaatgttgaaaaggattat<br>attgtcggatttaagtcaggagtgaaaaccgcatctgtcaaaaaggacatcatcaaagag<br>agcggcggaaaagtggacaagcagtttagaatcatcaacgcggcaaaagcgaagctagac<br>aaagaagcgcttaaggaagtcaaaaatgatccggatgtcgcttatgtggaagaggatcat<br>gtggcccatgccttggcgcaaaccgttccttacggcattcctctcattaaagcggacaaa<br>gtgcaggctcaaggctttaagggagcgaatgtaaaagtagccgtcctggatacaggaatc<br>caagcttctcatccggacttgaacgtagtcggcggagcaagctttgtggctggcgaagct<br>tataacaccgacggcaacggacacggcacacatgttgccggtacagtagctgcgcttgac<br>aatacaacgggtgtattaggcgttgcgccaagcgtatccttgtacgcggttaaagtactg<br>aattcaagcggaagcggatcatacagcggcattgtaagcggaatcgagtgggcgacaaca<br>aacggcatggatgttatcaatatgagccttgggggagcatcaggctcgacagcgatgaaa<br>caggcagtcgacaatgcatatgcaagaggggttgtcgttgtagctgcagcagggaacagc<br>ggatcttcaggaaacacgaatacaattggctatcctgcgaaatacgattctgtcatcgct<br>gttggtgcggtagactctaacagcaacagagcttcattttccagtgtgggagcagagctt<br>gaagtcatggctcctggcgcaggcgtatacagcacttacccaacgaacacttatgcaaca<br>ttgaacggaacgtcaatggcttctcctcatgtagcgggagcagcagctttgatcttgtca<br>aaacatccgaacctttcagcttcacaagtccgcaaccgtctctccagcacggcgacttat<br>ttgggaagctccttctactatgggaaaggtctgatcaatgtcgaagctgccgctcaataa	MMRKKSFWLGMLTALMLVFTMAFSDSASAAQPAKNVEKDYIVGFKSGVKT<br>ASVKKDIIKESGGKVDKQFRIINAAKAKLDKEALKEVKNDPDVAYVEEDH<br>VAHALAQTVPYGIPLIKADKVQAQGFKGANVKVAVLDTGIQASHPDLNVV<br>GGASFVAGEAYNTDGNGHGTHVAGTVAALDNTTGVLGVAPSVSLYAVKVL<br>NSSGSGSYSGIVSGIEWATTNGMDVINMSLGGASGSTAMKQAVDNAYARG<br>VVVVAAAGNSGSSGNTNTIGYPAKYDSVIAVGAVDSNSNRASFSSVGAEL<br>EVMAPGAGVYSTYPTNTYATLNGTSMASPHVAGAAALILSKHPNLSASQV<br>RNRLSSTATYLGSSFYYGKGLINVEAAAQ
P. aeruginosa IIVV-SD1 *las*B gene and LasB protein	atgaagaaggtttctacgcttgacctgttgttcgttgcgatcatgggtgtttcgccggcc<br>gcttttgccgccgacctgatcgacgtgtccaaactccccagcaaggctgcccagggcgcg<br>cccggcccggtcaccttgcaagccgcggtcggcgctggcggtgccgacgaactgaaagcg<br>atccgcagcacgaccctgcccaacggcaagcaggtcacccgctacgagcaattccacaac<br>ggcgtacgggtggtcggcgaagccatcaccgaagtcaagggtcccggcaagagcgtggcg<br>gcgcagcgcagcggccatttcgtcgccaacatcgctgccgacctgccgggcagcaccacc<br>gcggcggtatccgccgagcaggtgctggcccaggccaagagcctgaaggcccagggccgc<br>aagaccgagaatgacaaagtggaactggtgatccgcctgggcgagaacaacatcgcccaa<br>ctggtctacaacgtctcctacctgattcccggcgagggactgtcgcggccgcatttcgtc<br>atcgacgccaagaccggcgaagtgctcgatcagtgggaaggcctggcccacgccgaggcg<br>ggcggccccggcggcaaccagaagatcggcaagtacacctacggtagcgactacggtccg<br>ctgatcgtcaacgaccgctgcgagatggacgacggcaacgtcatcaccgtcgacatgaac<br>ggcagcaccgacgacagcaagaccacgccgttccgcttcgcctgcccgaccaacacctac<br>aagcaggtcaacggcgcctattcgccgctgaacgacgcgcatttcttcggcggcgtggtg<br>ttcaaactgtaccgggactggttcggcaccagcccgctgacccacaagctgtacatgaag<br>gtgcactacgggcgcagcgtggagaacgcctactgggacggcacggcgatgctcttcggc<br>gacggcgccaccatgttctatccgctggtgtcgctggacgtggcggcccacgaggtcagc<br>cacggctttaccgagcagaactccgggctgatctaccgcgggcaatcaggcggaatgaac<br>gaagcgttctccgacatggccggcgaggcagccgagttctacatgcgcggcaagaacgac<br>ttcctgatcggctacgacatcaagaagggcagcggtgcgctgcgctacatggaccagccc<br>agccgcgacgggcgatccatcgacaacgcgtcgcagtactacaacggcatcgacgtgcac<br>cactccagcggcgtgtacaaccgtgcgttctacctgttggccaattcgccgggctgggat<br>acccgcaaggccttcgaggtgttcgtcgacgccaaccgctactactggaccgccaccagc<br>aactacaacagcggcgcctgcggggtgattcgctcggcgcagaaccgcaactactcggcg<br>gctgacgtcacccgggcgttcagcaccgtcggcgtgacctgcccgagcgcgttgtaa	MKKVSTLDLLFVAIMGVSPAAFAADLIDVSKLPSKAAQGAPGPVTLQAAV<br>GAGGADELKAIRSTTLPNGKQVTRYEQFHNGVRVVGEAITEVKGPGKSVA<br>AQRSGHFVANIAADLPGSTTAAVSAEQVLAQAKSLKAQGRKTENDKVELV<br>IRLGENNIAQLVYNVSYLIPGEGLSRPHFVIDAKTGEVLDQWEGLAHAEA<br>GGPGGNQKIGKYTYGSDYGPLIVNDRCEMDDGNVITVDMNGSTDDSKTTP<br>FRFACPTNTYKQVNGAYSPLNDAHFFGGVVFKLYRDWFGTSPLTHKLYMK<br>VHYGRSVENAYWDGTAMLFGDGATMFYPLVSLDVAAHEVSHGFTEQNSGL<br>IYRGQSGGMNEAFSDMAGEAAEFYMRGKNDFLIGYDIKKGSGALRYMDQP<br>SRDGRSIDNASQYYNGIDVHHSSGVYNRAFYLLANSPGWDTRKAFEVFVD<br>ANRYYWTATSNYNSGACGVIRSAQNRNYSAADVTRAFSTVGVTCPSAL
