# Supplementary material for: Biodegradation of Pine Processionary Caterpillar Silk Is Mediated by Elastase- and Subtilisin-like Proteases
Source: Int J Mol Sci. 2022 Dec 3;23(23):15253. doi: 10.3390/ijms232315253 (PMC9741414; doi:10.3390/ijms232315253)
Supplement: Supplementary file 1 [file ijms-23-15253-s001.zip › Table S3-R1.pdf]

Supplementary Material. Table S3. List of proteases/peptidases deduced from the annotated genome of *P. aeruginosa* IIVV-SD1 sorted by their cellular location determined by *in silico* analysis and according to their homology with proteins present in databases (NCBI id). (EXT: extracellular; COM: cell outer membrane; CW: cell wall; PER: periplasmic space)

Number	Protein	Annotation	Cellular location	NCBI id
1	NODE_1_length_899382_cov_187.066277_563	Aeruginolysin (Alkaline metalloprotease)	EXT	Q03023.1
2	NODE_2_length_679634_cov_194.253327_356	Protease LasB (elastase, metalloendopeptidase)	EXT	P14756.1
3	NODE_2_length_679634_cov_194.253327_562	Extracellular serine protease (Ser-endopeptidase)	EXT	P09489.1
4	NODE_3_length_664803_cov_198.377296_440	Carboxy-terminal-processing protease (Ser-endopeptidase)	EXT	Q44879.1
5	NODE_5_length_443323_cov_188.588754_15	Secernin-2 (Cys-type exopeptidase)	EXT	Q96FV2.3
6	NODE_6_length_372047_cov_188.095835_4	Protease LasA (elastase, metalloendopeptidase)	EXT	Q02L18.1
7	NODE_8_length_305527_cov_190.385250_184	Aminopeptidase (metalloexopeptidase)	EXT	Q02PA2.1
8	NODE_12_length_204530_cov_195.990434_158	Lysyl endopeptidase (Ser- peptidase)	EXT	Q02SZ7.1
9	NODE_21_length_68114_cov_204.118177_73	Immunomodulating metalloprotease (metallopeptidase)	EXT	Q9I5W4.1
10	NODE_1_length_899382_cov_187.066277_297	Porin D (Ser-peptidase)	COM	P32722.1
11	NODE_3_length_664803_cov_198.377296_439	Murein hydrolase activator EnvC (metalloendopeptidase)	COM/PER	P37690.2
12	NODE_4_length_512579_cov_183.410353_265	Porin D (Ser-peptidase)	COM	P32722.1
13	NODE_4_length_512579_cov_183.410353_343	Porin D (Ser-peptidase)	COM	P32722.1
14	NODE_4_length_512579_cov_183.410353_373	Peptidyl-Asp metalloendopeptidase	COM	Q9I060.1
15	NODE_5_length_443323_cov_188.588754_300	Porin D (Ser-peptidase)	COM	P32722.1
16	NODE_7_length_323476_cov_190.432708_185	Tail-specific protease (Ser-endopeptidase)	COM/PER	P43669.2
17	NODE_10_length_241591_cov_199.467996_116	Porin D (Ser-peptidase)	COM	P32722.1
18	NODE_10_length_241591_cov_199.467996_141	Porin D (Ser-peptidase)	COM	P32722.1
19	NODE_13_length_202268_cov_195.422540_66	Alkaline phosphatase isozyme conversion prot (metallopeptidase)	COM-PER	P10423.1
20	NODE_20_length_68977_cov_198.681016_28	Porin D (Ser-peptidase)	COM	P32722.1
21	NODE_1_length_899382_cov_187.066277_523	Murein DD-endopeptidase MepH (Cys-type endopeptidase)	CW	P76190.1
22	NODE_1_length_899382_cov_187.066277_524	Murein DD-endopeptidase MepH (Cys-type endopeptidase)	CW	P76190.1
23	NODE_2_length_679634_cov_194.253327_604	Murein DD-endopeptidase MepH (Cys-type peptidase)	CW	P76190.1
24	NODE_3_length_664803_cov_198.377296_231	Lipoprotein NlpD/LppB homolog (metalloendopeptidase)	CW	P45682.1
25	NODE_3_length_664803_cov_198.377296_480	Zinc D-Ala-D-Ala carboxypeptidase (metallopeptidase)	CW	P00733.2
